# Bronchial sleeve resection for early-stage squamous cell carcinoma

**DOI:** 10.1186/1749-8090-7-33

**Published:** 2012-04-17

**Authors:** Taichiro Goto, Arafumi Maeshima, Kumi Akanabe, Ryoichi Kato

**Affiliations:** 1 Department of General Thoracic Surgery, National Hospital Organization Tokyo Medical Center, Tokyo, Japan; 2 Department of Pathology, National Hospital Organization Tokyo Medical Center, Tokyo, Japan; 3 Department of General Thoracic Surgery, National Hospital Organization Tokyo Medical Center, Meguro-ku, Tokyo, 152-8902, Japan

**Keywords:** Early-stage lung cancer, Bronchial sleeve resection, Surgery

## Abstract

A 75-year-old man complained of sputum and was referred to our department. His sputum cytology was class III. Chest X-ray and computed tomography showed no abnormalities, but bronchoscopy revealed an elevated lesion in the membranous portion of the left main bronchus, which was pathologically diagnosed as squamous cell carcinoma in situ. Since bronchoscopy revealed no other lesions in the visible parts of the airway, it was considered to be a solitary, early lung cancer, and sleeve resection of the left main bronchus was performed. The postoperative pathological diagnosis was squamous cell carcinoma in situ, pTisN0M0, stage 0. In recent years, an increasing number of studies have reported photodynamic therapy and brachytherapy for the treatment of early lung cancer. However, aggressive bronchoplastic surgery with emphasis on curability should be considered for lesions that are deemed resectable based on their number and extent of invasion.

## Background

As mass screening for lung cancer has become more widespread and bronchoscopy has advanced, the number of patients diagnosed with early lung cancer has increased [1]. However, the location and number of early lung cancers vary among the patients. Because of this and the diversification of therapeutic approaches, no standard treatment has been established to date. Early lung cancer is sometimes confined to the central portion at a distance from the lung parenchyma and, in such cases, curative treatment is possible by resecting the affected bronchus alone, while preserving the lung parenchyma. Herein, we report a patient who underwent sleeve resection of the left main bronchus for a centrally located, solitary, early lung cancer.

## Case report

**Figure 1:**
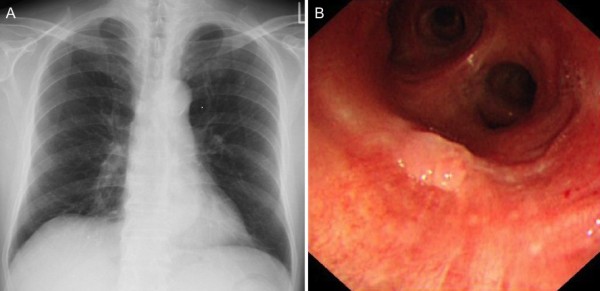
**Preoperative examination. a**, Chest X-ray showed no abnormalities. **b**, an elevated lesion was observed in the membranous portion of the left main bronchus, and was diagnosed as squamous cell carcinoma by bronchoscopic biopsy.

A 75-year-old man visited a nearby hospital with a chief complaint of sputum. His sputum cytology was class III, and he was referred to our department for further evaluation. He had a history of smoking 1 pack of cigarettes per day for 40 years. Chest X-ray and computed tomography showed no abnormalities (Figure 1a). Bronchoscopy revealed an elevated lesion in the membranous portion of the left main bronchus, which was pathologically diagnosed as squamous cell carcinoma by bronchoscopic biopsy (Figure 1b). Since bronchoscopy revealed no other lesions in the visible parts of the airway, we considered it to be a solitary, early lung cancer. Positron emission tomography showed no abnormal fluorodeoxyglucose uptake in the primary lesion or elsewhere in the body. Under a diagnosis of lung cancer (cTisN0M0), he underwent sleeve resection of the left main bronchus.

**Figure 2:**
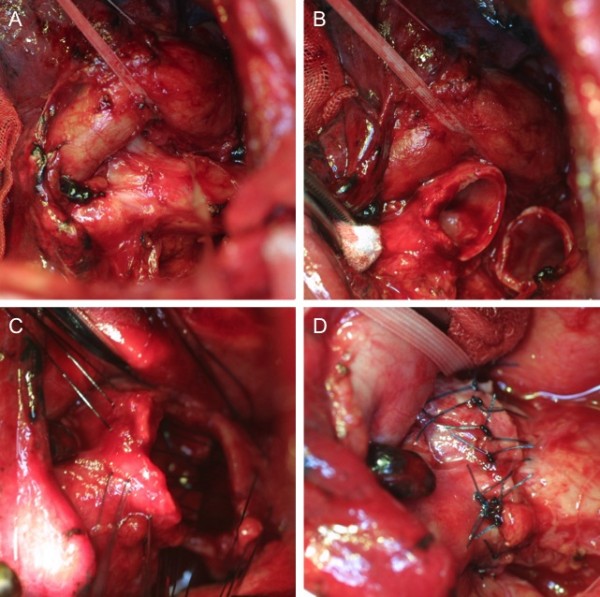
**Intraoperative photographs. a**, The left main pulmonary artery and left main bronchus were dissected and encircled with a vessel loop. **b**, Sleeve resection of the left main bronchus of 2 cartilages in length was performed. **c-d**, Interrupted absorbable sutures were placed circumferentially and knotted extraluminally

The surgical procedure was as follows. The visceral pleura was divided on the posterior aspect of the hilum to the level of the takeoff of the left upper lobe bronchus. The left main pulmonary artery was dissected circumferentially. Sub- and para-aortic lymph nodes were dissected. The left main bronchus was dissected circumferentially. Lower paratracheal, subcarinal, and hilar lymph nodes were dissected. The left main pulmonary artery and left main bronchus were encircled with a vessel loop and gently snared (Figure 2a). Frozen-section examination of the dissected lymph nodes showed no malignancy. Sleeve resection of the left main bronchus of 2 cartilages in length was performed (Figure 2b). The resected specimen was submitted for frozen-section examination, and both the proximal and distal margins were negative for cancer invasion. Interrupted sutures were placed circumferentially with absorbable monofilament 4–0 sutures so that the knots were outside of the bronchial lumen, and they were ligated (Figure 2c,d).

**Figure 3:**
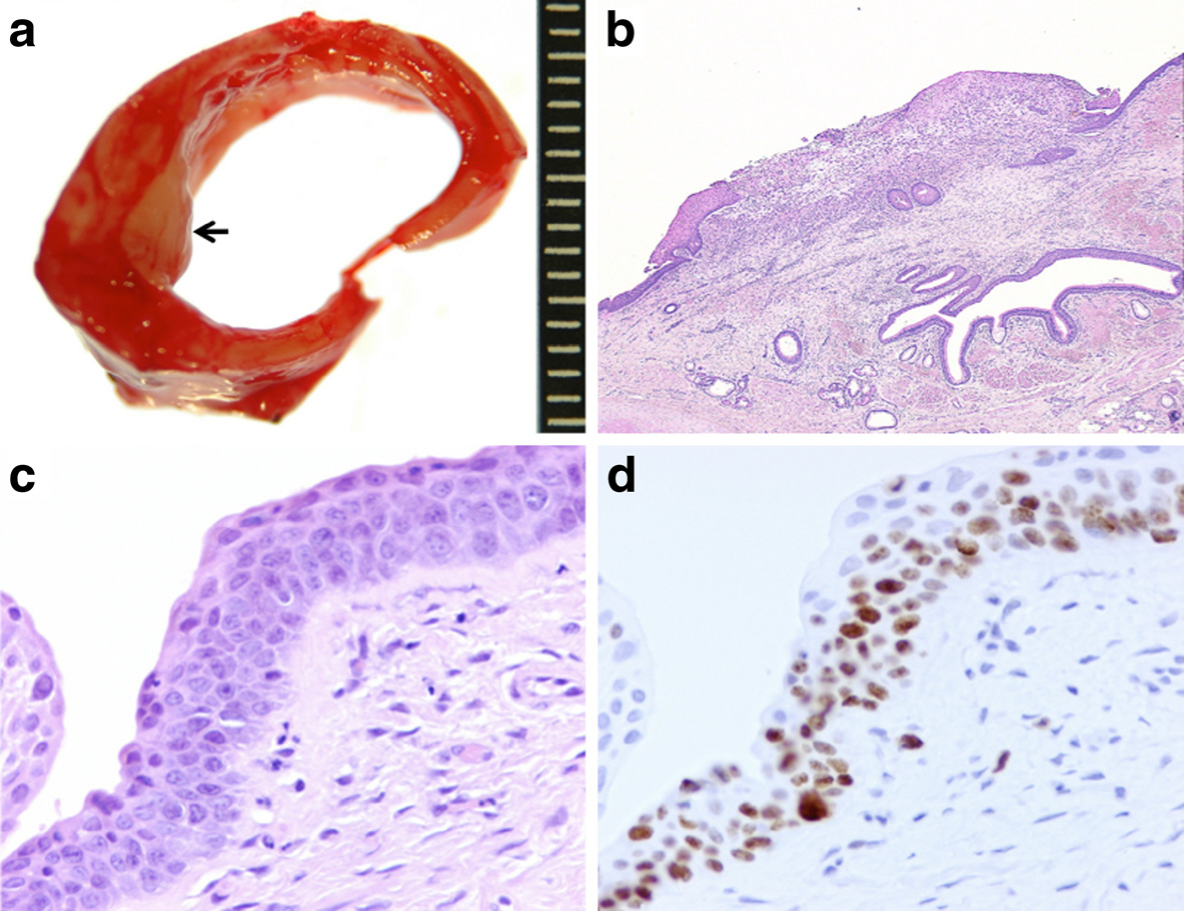
**Pathological findings. a**, Grossly, the tumor was a whitish, elevated nodule, as indicated by arrow. **b-c**, The elevated portion was composed of inflammatory granulation tissue and regenerated epithelium. Squamous cell carcinoma in situ were found throughout the entire thickness of the epithelium at the elevated margin. **d**, Almost all atypical cells were positive for Ki-67.

Grossly, the tumor was a whitish, elevated nodule (Figure 3a). Histologically, the elevated portion was composed of inflammatory granulation tissue and regenerated epithelium, and atypical squamous cells with large nuclei and conspicuous nucleoli were found in bronchial epithelium at the elevated margin (Figure 3b,c). The Ki-67-positive rate in the atypical squamous cell area was very high (Figure 3d). The atypical squamous cells did not invade beyond the basal membrane. These findings led to a diagnosis of squamous cell carcinoma in situ, pTisN0M0, stage 0.

**Figure 4:**
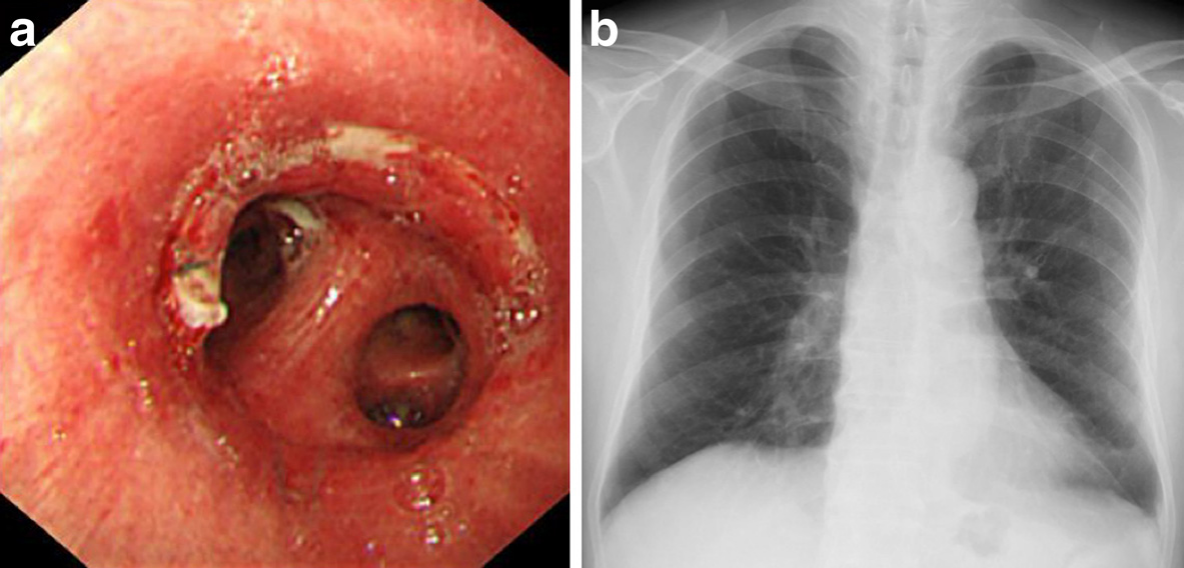
**Postoperative examination. a**, Postoperative bronchoscopy showed that the anastomosis was intact. **b**, Postoperative chest X-ray revealed good lung expansion bilaterally.

His postoperative course was uneventful. Postoperative bronchoscopy showed that the anastomosis was intact with no local dehiscence or stricture (Figure 4a). Chest X-ray showed good lung expansion (Figure 4b). After discharge, he was regularly followed-up by bronchoscopy in the outpatient clinic, but no recurrence at the suture line or new lesions in the airway have been detected. At present, 18 months after surgery, he remains free of disease.

## Discussions and conclusion

The Japan Lung Cancer Society (JLCS) has proposed their own sets of criteria for early lung cancer [2], although an internationally accepted set of criteria has not yet been determined. The JLCS defined early lung cancers as tumors that fulfill the following criteria: 1) chest X-ray findings are normal, 2) no lymph node or distant metastases are detectable using common methods for cancer staging, 3) the lesion is localized between the trachea and subsegmental bronchus, 4) the distal margin of the lesion is endoscopically visible, 5) the lesion is less than 2 cm in diameter, and 6) histologically, the lesion is squamous cell carcinoma [2]. Early lung cancer is located at sites that can be directly viewed through a bronchoscope; therefore, many surgeons recommend noninvasive treatments such as photodynamic therapy and endobronchial brachytherapy [3,4]. The patient’s poor general condition and presence of multiple lesions are good indications for these therapies [5]. However, these treatments presuppose accurate tumor staging, including lymph node metastasis, and their effects have not been verified. Moreover, medical equipment for these therapies is available in only a few centers, and the number of patients who can receive such treatments is currently very small. On the other hand, the main advantage of surgery is its optimal local control. In addition, surgery allows the accurate pathological evaluation of cancer invasion and presence of lymph node metastasis. Surgery also allows us to change surgical procedures based on the results of intraoperative frozen-section analysis and intraoperative findings, thereby increasing the curability. In recent years, advances in endobronchial ultrasonography have made it possible to histologically diagnose mediastinal lymph node metastases before surgery [6]. However, it remains difficult to definitively diagnose sub- and para-aortic lymph node metastases preoperatively. In the present patient, intraoperative pathological examination confirmed the absence of lymph node metastases, including the sub- and para-aortic lymph nodes.

Standard lobectomy or sleeve lobectomy is a usual surgical technique for early hilar lung cancer originating from lobar or segmental bronchi [7]. On the other hand, a lung-saving procedure is usually indicated for central tumors, for which the alternative is a pneumonectomy. It preserves normal lung tissue, reduces serious postoperative complications, and may enable the resection of cancer in selected patients with an inadequate reserve [8]. Many patients with early lung cancer are heavy smokers, frequently complicated by obstructive ventilatory disturbance, and develop multiple lung cancers synchronously or metachronously [9]. Surgery preserving the lung function as much as possible seems to be useful in terms of the quality of life and possibility of re-operation [10].

In conclusion, we report a case of early lung cancer arising from the left main bronchus, in which sleeve resection of the left main bronchus was performed. We believe that this procedure allows the curative resection of cancer while preserving the lung function. For the early detection of a second incidence of primary hilar tumor, repeated sputum cytology and bronchoscopy will be mandatory [11].

## Consent

Written informed consent was obtained from patient for publication of this case report and any accompanying images. A copy of the written consent is available for review by the Editor-in-Chief of this journal.

## Competing interests

The authors declare that they have no competing interests.

## Authors’ contributions

TG wrote the manuscript. TG, KA, and RK performed surgery. AM carried out the pathological examination. RK was involved in the final editing. All authors read and approved the final manuscript.
